# Safety and efficacy of arteriovenous fistula angioplasties performed by nephrologists: report from a Brazilian interventional nephrology center

**DOI:** 10.1590/2175-8239-JBN-2021-0085

**Published:** 2021-11-10

**Authors:** Ricardo P. Franco, Miguel C. Riella, Domingos C. Chula, Marcia T. de Alcântara, Marcelo M. do Nascimento

**Affiliations:** 1 Pro-Renal Brasil, Centro de Nefrologia Intervencionista, Curitiba, PR, Brasil.; 2 Faculdade Evangélica Mackenzie do Paraná, Curitiba, PR, Brasil.; 3 Universidade Federal do Paraná, Curitiba, PR, Brasil.

**Keywords:** Arteriovenous Fistula, Fistula, Nephrology, Angioplasty, Angioplasty, Balloon, Endovascular Procedures., Fístula Arteriovenosa, Fístula, Nefrologia, Angioplastia, Angioplastia com Balão, Procedimentos Endovasculares

## Abstract

**Introduction:**

Arteriovenous fistulas (AVF) are the first choice vascular access for hemodialysis. However, they present a high incidence of venous stenosis leading to thrombosis. Although training in interventional nephrology may improve accessibility for treatment of venous stenosis, there is limited data on the safety and efficacy of this approach performed by trained nephrologists in low-income and developing countries.

**Methods:**

This study presents the retrospective results of AVF angioplasties performed by trained nephrologists in a Brazilian outpatient interventional nephrology center. The primary outcome was technical success rate (completion of the procedure with angioplasty of all stenoses) and secondary outcomes were complication rates and overall AVF patency.

**Findings:**

Two hundred fifty-six angioplasties were performed in 160 AVF. The technical success rate was 88.77% and the main cause of technical failure was venous occlusion (10%). The incidence of complications was 13.67%, with only one patient needing hospitalization and four accesses lost due to the presence of hematomas and/or thrombosis. Grade 1 hematomas were the most frequent complication (8.2%). The overall patency found was 88.2 and 80.9% at 180 and 360 days after the procedure, respectively.

**Conclusion:**

Our findings suggest that AVF angioplasty performed by trained nephrologists has acceptable success rates and patency, with a low incidence of major complications as well as a low need for hospitalization.

## RESUMO

**Introdução:**

As fístulas arteriovenosas (FAV) são a primeira escolha de acesso vascular para hemodiálise. No entanto, elas apresentam uma alta incidência de estenoses venosas levando à trombose. Embora o treinamento em nefrologia intervencionista possa melhorar a acessibilidade para o tratamento das estenoses venosas, há dados limitados sobre a segurança e a eficácia desta abordagem realizada por nefrologistas treinados em países em desenvolvimento e de baixa renda.

**Métodos:**

Este estudo apresenta os resultados retrospectivos de angioplastias de FAV realizadas por nefrologistas treinados em um centro ambulatorial brasileiro de nefrologia intervencionista. O desfecho primário foi a taxa de sucesso técnico (conclusão do procedimento com angioplastia de todas as estenoses) e os desfechos secundários foram taxas de complicação e a patência geral das FAV.

**Achados:**

Duzentas e cinquenta e seis angioplastias foram realizadas em 160 FAV. A taxa de sucesso técnico foi de 88,77% e a principal causa de falha técnica foi a oclusão venosa (10%). A incidência de complicações foi de 13,67%, com apenas um paciente necessitando de internação e quatro acessos perdidos devido à presença de hematomas e/ou trombose. Hematomas de grau 1 foram a complicação mais frequente (8,2%). A patência geral encontrada foi de 88,2 e 80,9% a 180 e 360 dias após o procedimento, respectivamente.

**Conclusão:**

Nossos achados sugerem que a angioplastia de FAV realizada por nefrologistas treinados tem taxas de sucesso e patência aceitáveis, com uma baixa incidência de complicações maiores, bem como uma baixa necessidade de hospitalização.

## INTRODUCTION

According to the 2019 Brazilian Dialysis Census, approximately 140,000 patients are dependent on hemodialysis (HD) in Brazil and the number of incident patients has been steadily rising over the past decade^1^. Vascular access (VA) is an essential component of HD treatment, and the native arteriovenous fistula (AVF) still the first choice of VA for most incident HD patients given its lower morbidity, low cost, and long term patency compared to grafts and central venous catheters^2^.

Despite these well-known advantages, the AVF often develops venous stenosis (VS), which may lead to several signs and symptoms of VA dysfunction such as: difficulty in cannulation, low dialysis adequacy, formation of aneurysms, and prolonged bleeding after removal of the dialysis needles. VS also leads to low flow, thrombosis, and loss of the VA. In most of these cases, angioplasty is the best treatment option; however, this procedure is not broadly available in the Brazilian Public Health System, since it demands hospitalization in a high complexity cardiovascular center, as well as availability of a trained vascular surgeon.

In the United States of America (USA), AVF angioplasties are commonly performed by nephrologists in specialized outpatient centers, with lower complication rates, shorter hospital stay, lower costs, and fewer infection episodes compared to inpatient treatment. However, in developing countries, most nephrologists are not trained to perform this type of interventional procedure, which might affect AV outcomes^3^^-^ 6. An important obstacle in these countries and in low-resource settings is the lack of training in endovascular procedures for nephrologists.

Although it is believed that a proper training in interventional nephrology and endovascular procedures in outpatient VA centers may improve the accessibility to treatment for VA dysfunction, there is no data addressing the safety and efficacy of this approach in Brazil, and few data from developing countries or low resource settings. In this study, we present data regarding the success, complications, and patency rates of AVF subjected to angioplasty performed by trained nephrologists in a Brazilian outpatient surgical center.

## METHODS

This retrospective study assessed success, complication, and patency rates of AVF subjected to angioplasty by trained nephrologists in an outpatient surgical center.

We analyzed angioplasties performed in native AVF by interventional nephrologists from March 2014 to January 2018 at Pro Renal Foundation Brazil. Inclusion criteria were:

1. Endovascular procedure performed by a trained nephrologist for diagnosis or treatment of VA dysfunction.
2. Procedures performed in patients with a currently in use AVF or a maturing native AVF.

Exclusion criteria were:

1. Procedures performed by vascular surgeons;
2. Procedures performed in patients with arteriovenous grafts;
3. Procedures performed in patients using exclusively a central venous catheter;
4. Procedures performed in patients not currently on HD (i.e. transplant or PD patients);
5. Angiographies performed during central venous catheter insertion for diagnostic purposes.

Patients from 4 HD facilities with approximately 600 patients were referred to the center with signs or symptoms of AVF dysfunction. VS were confirmed by Doppler ultrasound, and patients were referred for angioplasty if they had a stenosis greater than 50% associated with clinical or functional manifestations of AVF dysfunction, such as difficulty in cannulation, inability to maintain HD prescribed blood flow, prolonged bleeding after needle removal, urea Kt/V below 1.2, intra-access blood flow below 600 mL/min on Doppler or a decrease greater than 25% in relation to previous measurements. Doppler ultrasound was also performed by a nephrologist. All patients were informed of the risks and benefits of the procedures and agreed to sign an informed consent form.

### ANGIOPLASTY TECHNIQUE AND MATERIALS

AVF venipuncture was performed for access, followed by contrast injection (Omnipaque, GE Healthcare, USA) in order to obtain a digital subtraction phlebography through an OEC 9900 C-arm (General Electric, USA). Intraoperative ultrasound (LOGIQe, General Electric, USA) was performed at the discretion of the interventionist to identify complications and device positioning. Conscious sedation was achieved with intravenous midazolam with or without fentanyl, administered in a titrated dose to achieve moderate sedation and analgesia and avoid airway and spontaneous ventilation impairment. Patients were kept on nasal supplemental oxygen and monitored throughout the procedure for heart rhythm, heart rate, blood pressure, oxygen saturation, and level of consciousness. All personnel involved in the procedure wore radiation protective equipment, and radiation exposure levels were measured according to local regulation requirements. Materials available were standard vascular introducer sheaths, ZipWire and V-18 guidewires, Imager II diagnostic catheters, and Mustang high pressure balloons (Boston Scientific, USA). Eighty-three cases were treated with a Cutting Balloon (Boston Scientific, USA), because patients were enrolled in an ongoing randomized controlled trial.

Stenoses greater than 50% were treated by angioplasty with a balloon with a diameter 10-20% larger than the normal adjacent vein. Balloons were inflated in the VS area until the resolution of the balloon waist or the nominal inflation pressure was reached.

After the angioplasty, a new image was obtained to rule out complications and evaluate residual VS. All patients were observed for up to 2 hours before discharge.

The follow-up was carried out in an outpatient setting with Doppler ultrasonography within 3 weeks from the procedure and then quarterly, until loss of the access, transplantation, or change to peritoneal dialysis.

### OUTCOMES

The primary outcome was technical success rate of the intervention, defined as completion of the procedure with angioplasty of all stenoses in the AVF circuit. Secondary outcomes were complications rate and overall patency of AVF.

We also evaluated the individual radiological success of lesions subjected to angioplasty, which is defined as a residual stenosis of less than 30% after the dilation and the association of independent variables with radiological procedure failure, such as patient gender, age, presence of diabetes or coronary artery disease, location of the stenosis, type and age of the AVF, and time elapsed since the nephrologist's training.

Complications were graded according to the classification proposed by the American Society of Diagnostic and Interventional Nephrology (ASDIN)^7^. Grades are applied to each type of complication (hematomas, venous rupture, arterial complications, hypotension, and respiratory complications) considering its specific presentation, possible treatments, and outcomes. Grade 1 complications require only nominal therapy with no need for specific treatment or increase in level of care and have no clinical consequence. Grade 2 require minor or percutaneous therapy, with no significant long term sequelae. Grade 3 complications require major therapy, surgical repair, or hospitalization. Grade 4 complications imply long-term sequelae, loss of function, or death. Grade 1 hematomas related to puncture and minor venous rupture during dilation were recorded as the same complication on the database, so their frequency is summed in the analysis.

The overall AVF patency was defined as the duration of the access survival, with or without new interventions, until the occurrence of thrombosis without the possibility of salvage or VA abandonment. Patients who died, underwent transplantation or migrated to peritoneal dialysis, were censored. Patients with venous occlusions that precluded guidewire crossing and angioplasty were considered technical failures and were not included in the patency analyses, since they did not actually receive angioplasty and access patency would not represent an effect of treatment.

### STATISTICAL ANALYSIS

The data regarding accesses, such as its creation date, lesion grading, outcome and procedure-related complications, were recorded immediately after each procedure, using electronic forms and the database of the center's VA program and tabulated for retrospective analysis. All statistical analysis were performed with the R software. The Shapiro test was performed to assess the normality of the variables.

Multivariate analysis by logistic regression was used to assess the association between patient characteristics, AVF type and age, stenoses site and time elapsed from the nephrologist's training and angioplasty radiological failure. The 95% confidence interval is provided for each outcome. Values of p below 0.05 were considered significant.

The AVF survival was analyzed using Kaplan-Meier method^8^. Thrombosis, abandonment, or loss of the AVF for any reason was considered as an event. Deaths, transplantations, changes to peritoneal dialysis, or losses of follow-up were censored. The percentage of patent accesses was analyzed at 180, 360, and 720 days after the first procedure. Log-Rank was used to compare between-group survival differences regarding the presence or absence of diabetes, coronary artery disease, hypertension, patient gender, different age groups, distal and proximal locations of the access, and AVF age. Multivariate analysis by the Cox Proportional Hazards test was performed to assess the correlation of the aforementioned variables and access survival.

## RESULTS

One hundred fifty-five patients with 160 AVF underwent angioplasty. The characteristics of patients and VA are described in Table 1. Two hundred fifty-six angioplasties were performed and 29 unsuccessful attempts occurred due to venous occlusions, which precluded guidewire crossing and, thus, the treatment. No complications occurred during attempts to cross the lesions in cases with venous occlusion.

**Table 1:** Characteristics of patients and vascular accesses

Pacientes (N=155)			Accesses (N=160)	
Age (years)				
		58.5 (± 14)	Age (days)	574.2 (± 615.2)
Male (%)		65.6	Age less than 3 months (%)	16.2
Diabetes (%)		44.4	Radiocephalic (%)	28.7
Hipertension (%)		80.1	Brachiocephalic (%)	38.1
CAD (%)		17.2	Brachiomedian	22.5
			Brachiobasilic (%)	10.6

Patient and AVF ages shown as mean± SD. CAD: Coronary Artery Disease

The technical success rate was 88.77%, and the venous occlusions were the main cause of technical failure (10.18%). In 3 cases, the procedure was interrupted due to complications (1.05%), consisting of an unintended arterial puncture, a perforation of a collateral vein in the chest during an attempt to cross a high grade stenosis in the innominate vein, and a thrombosis in an unused AVF, with no possibility of salvage. There was no need for hospitalization in any of these cases. Five accesses (1.75%) could not be used for HD after the angioplasty due to grade 4 hematomas, thrombosis, or cannulation impossibility.

Multiple stenosis were found in 44.62% of the AVF and a total of 383 lesions were treated, with a radiological success in 303 (79.11%). In 75 of the 256 cases that underwent angioplasty (29.29%), there was at least one lesion resistant to angioplasty.

Cephalic arch stenoses were present in 10.54% of the procedures and central vein stenoses were present in 22.26%. Logistic regression (Table 2) showed that thes stenosis sites were related to a higher probability of radiological failure (p=0.004 and p=0.001 respectively). Gender, age, comorbidities, age of AVF, and time elapsed between the nephrologist's training and the procedure were not significantly associated with radiological failure, both in univariate or multivariate analyses (data not shown).

**Table 2:** Multivariate logistic regression for factors associated with angioplasty radiological failure (N=256)

	OR (95%CI)	P
Juxta-anastomotic stenosis	1.566 (0.616 – 3.979)	0.988
Cephalic arch stenosis	5.907 (2.098 – 17.803)	0.004
Central vein stenosis	6.06 (2.164 – 18.097)	0.001
Distal AVF	1.086 (0.491 – 2.340)	0.990

## COMPLICATIONS

Complications occurred in 13.67% of the cases (35 out of 256, excluding 29 occlusions that were not submitted to angioplasty and did not present complications while trying to cross the occlusions).

Grade 1 hematomas due to puncture or minor contrast extravasation after balloon dilation were the most frequent complication (8.20%), and did not require treatment or decreased access blood flow. Two patients (0.8%) presented Grade 2 hematomas with VA blood flow reduction and needed manual compression for controlling the hematoma.

Four patients evolved with permanent loss of the VA (1.56%), two because of thrombosis of low flow accesses with resistant stenoses and another two due to Grade 4 hematomas and vein rupture after angioplasty. All of these VA had symptoms, difficulty or impossibility of cannulation or low flow on Doppler Ultrasound. One case of thrombosis in a radiocephalic AVF due to transient hypotension during angioplasty was successfully treated with 2 mg alteplase and angioplasty of the stenoses (Grade 2 complication).

One unintended brachial artery puncture occurred while accessing a severely swollen arm due to brachycephalic vein occlusion, which required only manual compression (Grade 1).

Six cases (2.3%) of clinical complications happened during the procedures: two transitory hypotensions, with one needing infusion of fluids (Grade 2), one hypoxemia with need for increase in supplemental oxygen (Grade 2), one mild reaction to radiocontrast managed with hydrocortisone, and one case of bacteremia after removal of an asymptomatic infected catheter during the angioplasty, which needed hospitalization (Grade 3).

The multivariate logistic regression showed no significant impact of the independent variables on complication occurrence (data not shown).

Two deaths occurred within 30 days of the procedures (0.78%). The first patient was a 70-year-old woman who had already undergone 4 cephalic arch and central angioplasties within one year, all without complications, and who had been on HD for ten years. She died 20 days after an angioplasty, probably due to acute myocardial infarction with a report of chest pain and dyspnea on the day before HD. The second patient, a 73-year-old woman who was on renal replacement therapy for 5 years, presented a sudden illness followed by cardiorespiratory arrest 12 days after an uneventful angioplasty procedure.

### ACCESS PATENCY

**Figure 1:**
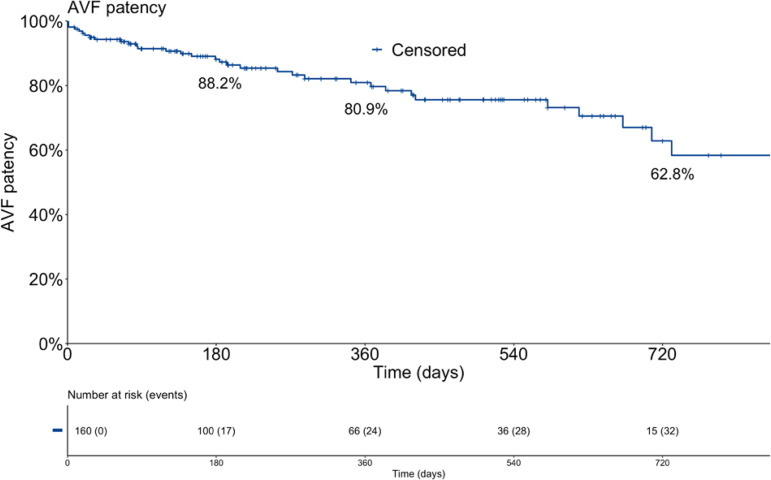
Kaplan-Meier curve of vascular access overall patency in days after the procedure.

The overall AVF patency assessed with Kaplan-Meier analysis was 88.2, 80.9, and 62.8% in 180, 360 and 720 days after the procedure, respectively (Figure 1). Follow-up time ranged from 8 to 1406 days (354±308).

**Figure 2:**
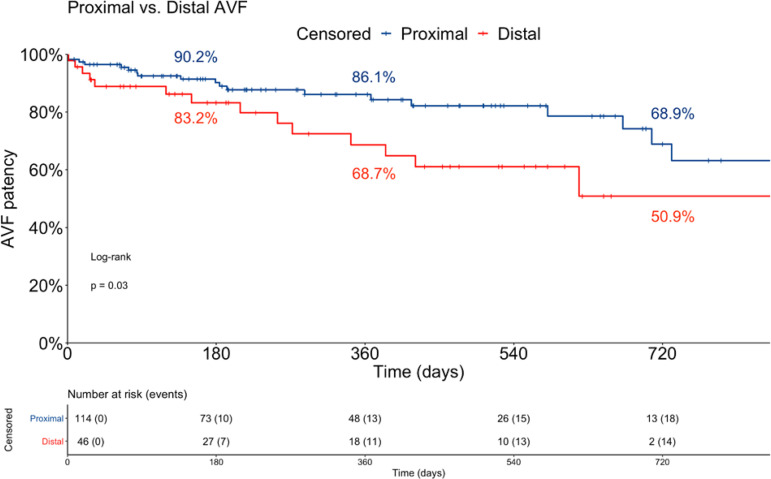
Comparison of survival of distal versus proximal arteriovenous fistulas (AVF) after the procedure.

Distal AVF showed less patency compared to proximal ones in the assessment of factors correlated with survival, as shown in Figure 2. Gender, age, AVF age, coronary artery disease, diabetes mellitus, and hypertension did not significantly impact VA patency during follow-up.

Moreover, in the multivariate analysis by the Cox Proportional Hazards test, distal AVF showed a significantly higher risk of access-related events and AVF failure (HR 2.14, 95% CI 1.02 - 4.47, p=0.0421) compared to proximal AVF. Coronary artery disease, diabetes, hypertension, and age group did not have a significant impact in access patency (data not shown).

## DISCUSSION

In the present study, AVF angioplasty performed in an outpatient setting by trained nephrologists showed a technical success rate of 88.77%, and venous occlusions were the most frequent cause of procedure failure, followed by 3 interruptions due to complications. Radiological failure was significantly more frequent in cases with cephalic arch or central veins stenoses. Radiological success was reached in 79% of the treated VS.

Four access-related complications led to loss of the VA (1.6%) and one patient required hospitalization (0.4%). AVF patency within one year from the procedure was 80.9%, and we observed a significantly higher survival in proximal AVF.

The technical success rate and low incidence of serious complications in this study suggest that most AVF stenoses can be treated with efficacy and safety using an endovascular approach in an outpatient setting.

Venous occlusions, which prevent the passage of the guidewire and dilation of the stenotic lesions, were the major cause of failure (10%). The literature shows similar rates of immediate success, ranging from 84 to 96%^3^^,^ 9 ^-^ 11. The overall patency within one year was 80.9%, which is comparable to other studies that show one-year survival ranging from 61 to 96% 12 ^-^ 20. It is possible that technical failures would be less if different guidewires and catheters were available to facilitate the management of critical stenoses and occlusions. Also, the experience of the interventionist plays an important role in these cases, and the data includes our team learning curve and first procedures.

Complications occurred in 13.67% of the angioplasties, with the majority requiring only nominal therapy, classified as Grade 1 by the ASDIN classification.^7^ Other studies show complication rates ranging from 4.48 to 7% in procedures performed by nephrologists^3^^,^ 21 ^,^ 22, with major complications in 0.19 to 1.7%. Our series showed four VA losses after angioplasty and two deaths within 30 days of procedures, a total of 2.3% of major complications. However, we believe that the interventions were justified as the two accesses lost due to grade 4 hematomas had low flow and symptoms of VA dysfunction and the two lost due to thrombosis had resistant lesions that were already causing symptoms. Also, we believe it's very unlikely that the two deaths were related to the procedures, considering there were no procedure-related complications and the long dialysis vintage of the patients.

In our study, grade 1 hematomas were the most frequent complication (8.20%). We believe that including puncture-related hematomas in this category, in addition to those related to balloon dilation, partially explains the higher incidence of this complication, since many studies include only dilation-related hematomas during angioplasty.

The low rate of major complications and hospitalizations are important data for safety assessment of performing these procedures in an outpatient setting. The participation of nephrologists in the planning, creation, and intervention of the vascular accesses has been described in several countries with good results^3^^,^ 23 ^,^ 24.

Percutaneous angioplasty is considered the first choice treatment for stenosis in AVF^25^, despite the high rate of restenosis and reinterventions^26^. The performance of these procedures in an outpatient setting potentially reduces costs compared to inpatient treatment, with no increase in risks or complications. Brenner et al. retrospectively compared the costs of inpatient and outpatient approaches for 898 patients with VA dysfunction. The values associated with inpatient and outpatient treatment per patient were US $ 8,265 and US $ 1,491, respectively, a 72% lower cost^27^. Another retrospective study correlated the creation of a VA center with a reduction of up to 0.64 days of hospitalization per patient/year, as well as 0.31 missed appointments per patient/year^4^. Furthermore, the creation of interventional nephrology centers can decrease the use of catheters, hospitalizations, costs, and missed dialysis sessions, as well as increase AVF rates^3^^,^ 4 ^,^ 28.

Traditionally, the procedures for the diagnosis and treatment of VA dysfunction are performed by vascular surgeons and interventional radiologists; however, in recent years and in several countries, interventional nephrologists have performed these procedures safely and with good results^3^^,^ 4 ^,^ 28.

Other authors have published experiences on the role of interventional nephrologists regarding vascular accesses in developing countries^29^^,^ 30. These reports have in common the decrease in the waiting time for the procedures and the higher accessibility of patients to treatment. The dialysis population is growing and the need for VA planning and maintenance puts our health care model to the test.

Many Brazilian regions do not have hospitals with adequate infrastructure and sufficient number of trained professionals to manage these cases. The inclusion of the nephrologist in the management of VA and the implementation of outpatient treatments can provide adequate care for HD patients and reduce costs with good results, as presented in this study.

Initiatives such as the Educational Ambassadors Program of the International Society of Nephrology, which was responsible for training in our unit, could spread the development of interventional nephrology, and thus promote accessibility for patients to previously unavailable treatments in developing countries. The training led us to develop a structured VA program with three interventional nephrologists, two vascular surgeons, one nurse, and one VA technician, with one interventional nephrologist being responsible for access coordination and surveillance using Doppler ultrasound. In the past, our patients did not have access to angioplasty, resulting in abandonment of VA due to symptoms or thrombosis. In our experience, the results of this training went further since our center has become a national reference in interventional nephrology, providing periodic training for doctors from Brazil and South America.

This study had the limitations inherent of a retrospective study carried out in a single center, but the use of a prospective database, filled immediately after each procedure may mitigate some of the biases related to retrospective reviewing of health records and reduce reporting errors. In addition, only three nephrologists were trained and the results cannot be extrapolated to other physicians concerning familiarity with interventional procedures.

Our findings suggest that AVF angioplasty performed in the outpatient setting by trained nephrologists has acceptable success and patency rates and a low frequency of major procedure-related complications and hospitalization, in line with the current literature. Training programs may improve patients' accessibility to this treatment, especially in developing countries.
